# High prevalence of gastroesophageal reflux symptoms in type 2 diabetics with hypoadiponectinemia and metabolic syndrome

**DOI:** 10.1186/1743-7075-9-4

**Published:** 2012-01-25

**Authors:** Ayumu Hirata, Ken Kishida, Hideaki Nakatsuji, Kana Inoue, Aki Hiuge-Shimizu, Tohru Funahashi, Iichiro Shimomura

**Affiliations:** 1 Department of Metabolic Medicine, Graduate School of Medicine, Osaka University, Suita, Osaka 565-0871, Japan; 2 Department of Metabolism and Atherosclerosis, Graduate School of Medicine, Osaka University, Suita, Osaka 565-0871, Japan

**Keywords:** gastroesophageal reflux symptom, metabolic syndrome, visceral fat, adiponectin

## Abstract

**Abstract:**

**Background**

The prevalence of gastroesophageal reflux disease (GERD) has been increasing worldwide. Abdominal obesity or visceral fat accumulation rather than simple obesity is associated with GERD. Previous reports demonstrated the association between GERD and type 2 diabetes mellitus (T2DM). Signification of visceral fat accumulation and adiponectin in T2DM patients with GERD remains unclear. The present study investigated the relationships between GERD symptoms, visceral fat accumulation and adiponectin in subjects with T2DM.

**Findings**

The study (ADMIT study) subjects were 66 Japanese T2DM outpatients, who answered the questionnaire regarding GERD symptoms in Frequency Scale for the Symptoms of GERD (FSSG), and were measured visceral fat area by bioelectrical impedance analysis. Patients with FSSG scores of more than 8 were considered as positive. The prevalence of FSSG score ≥ 8 and average FSSG score in T2DM subjects with the metabolic syndrome (Mets) were significantly higher compared to those without Mets. The prevalence of FSSG score ≥ 8 and average FSSG score in T2DM subjects with low levels of serum adiponectin were significantly higher compared to those with high levels of serum adiponectin. Moreover, the combination of Mets and hypoadiponectinemia had a multiplicative effect on GERD symptom score (p = 0.047).

**Conclusions**

Our study showed that the coexistence of MetS and low levels of serum adiponectin was associated with the higher prevalence of FSSG score ≥ 8 and the higher scores of GERD symptom in subjects with T2DM.

**Trial Registration:**

UMIN 000002271.

## Background

The prevalence of gastroesophageal reflux disease (GERD) has been increasing worldwide [1], and is associated with impairment of quality of life (QOL), Barrett's esophagus, esophageal carcinogenesis and lifestyle-related diseases including sleep dysfunction, metabolic disorders and heart disease [2]. This increase is likely associated with the increased prevalence of obesity [2]. Obesity is shown to be an independent risk factor of GERD [3], through increased transient lower esophageal sphincter relaxation, which is an important mechanism of GERD [4]. Abdominal obesity or visceral fat accumulation rather than simple obesity is associated with GERD [5,6]. With accumulation of visceral fat, circulating levels of adiponectin, a potential anti-inflammatory adipocytokine, are decreased, whereas circulating levels of proinflammatory cytokines, such as interleukin-6 (IL-6), are increased. Low serum adiponectin levels were reported in subjects with esophago-gastro-duodenoscopic erosive esophagitis [7] or obese patients with GERD [8,9]. Visceral fat accumulation and dysregulated production of adipocytokines could exacerbate local inflammation at the esophagogastric junction.

Previous reports demonstrated the association between GERD and type 2 diabetes mellitus (T2DM) in Japanese [10-12]. Signification of visceral fat accumulation and adiponectin in T2DM patients with GERD remains unclear. Ambulatory 24-h pH test-monitoring is the gold standard for diagnosing GERD [13]. This method, however, places pressure on patients. A questionnaire for the diagnosis of reflex disease (QUEST) was introduced and its usefulness for GERD diagnosis has been evaluated in Western countries [14]. However, QUEST have several problems that it was not easy to complete the questionnaire and evaluate therapeutic response of GERD in Japanese [15], and therefore, a new questionnaire for Japanese (FSSG; Frequency Scale for the Symptoms of GERD) was developed [16]. The present study investigated the relationships between GERD symptom score using FSSG, visceral fat accumulation and adiponectin in Japanese T2DM patients.

## Methods and Procedures

### Study population

Subjects were recruited from consecutive Japanese T2DM outpatients, who answered the questionnaire regarding GERD symptoms in FSSG, and were measured visceral fat area by bioelectrical impedance analysis (BIA) [17], in the "Diabetes & Metabolic Station", Osaka University Hospital, between September 2009 and November 2011. Exclusion criteria included patients that were pregnant, a nursing mother, or a shift worker. Disease-related exclusion criteria included the following; malignant diseases, active peptic ulcer disease, or a past history of upper gastrointestinal surgery. Patients were also ineligible if they had received continuously acid-suppression drugs (histamine-2 blockers and proton-pump inhibitors). The study subjects comprised 66 Japanese excluding subjects who were treated with pioglitazone (n = 4), which is known to increase serum adiponectin levels in T2DM [18]. This study approved by the Medical Ethics Committee of Osaka University. All participants were Japanese and each gave a written informed consent. This study (ADMIT study) is registered under number UMIN 000002271.

https://upload.umin.ac.jp/cgi-open-bin/ctr/ctr.cgi?function=brows&action=brows&type=summary&recptno=R000002777&language=E

### GERD score

The FSSG consisted of 12 questions, which were scored to indicate the frequency of symptoms as follows: never = 0; occasionally = 1; sometimes = 2; often = 3; and always = 4. Patients with FSSG scores of more than 8 were considered as positive (When the cut-off score was set at 8 points, this test shows a sensitivity of 62%, a specificity of 59%, and an accuracy of 60% [16]). Based upon 80% power to detect statistically significant differences (p = 0.05; two-sided), a sample size of at least 25 patients in each group was required to demonstrate (total sample size = 50), according to previous reports [9,10].

### Anthropometry and laboratory measurements

Venous blood samples were collected in the morning after overnight fast for measurements of each parameter and adiponectin (Otsuka Pharmaceutical Co., Tokushima, Japan, intra-coefficient of variation (CV); < 10%, inter-CV < 10%), IL-6 (Human IL-6 Quantikine ELISA Kit, R&D Systems, USA, intra-CV; 7.3%, inter-CV 7.7%), as we previously reported [18,19]. Serum concentration of thiobarbituric acid-reacting substance (TBARS), an important biomarker of systemic oxidative stress reflecting serum lipid peroxidation products, was determined by the method of Yagi (Japan Institute for the Control of Aging, Nikken SEIL Co., Shizuoka, Japan), as reported previously by our group [18]. Diabetic retinopathy, nephropathy and peripheral neuropathy were diagnosed, as we previously reported [20]. The metabolic syndrome (Mets) was diagnosed according to the Japanese guidelines for Mets based on VFA ≥ 100 cm^2 ^[21]. Briefly, the voltage recorded at the flank to the flow of current between the umbilicus and the back correlates significantly with visceral fat area (VFA) and is not influenced by subcutaneous fat. We reported previously that VFA estimated by BIA (eVFA) correlates significantly with that determined by computed tomography (CT) (r = 0.88, p < 0.0001) [17]. The CV of BIA with the value of CT was 0.89% in the standing position and late exhalation. A cutoff value for BIA-measured eVFA was 100 cm^2 ^for both males and females as criteria to screen coming multiple obesity-related cardiovascular risk factors [22].

### Statistical analysis

Data are mean ± SEM, and compared by the χ^2 ^and Mann-Whitney U-test in experiments of two groups. In all cases, a *p *value < 0.05 was considered statistically significant. All analyses were performed with the JMP Statistical Discovery Software 9.0 (SAS Institute, Cary, NC).

## Results

### Characteristics of subjects enrolled in the present study

The baseline characteristics of the patients are listed in Table 1. The prevalence of FSSG score ≥ 8 in subjects with T2DM was 23% (n = 15/66).

**Table 1:** Baseline characteristics of type 2 diabetic patients in the present study (n = 66)

	Metabolic syndrome (-) (n = 38)	Metabolic syndrome (+) (n = 28)	p value
Gender, male/female	18/20	9/19	0.210
Age, years	63 ± 1 (36-78)	67 ± 2 (40-88)	0.101
Body mass index, kg/m^2^	23.2 ± 0.53 (19.1-32.7)	25.9 ± 0.62 (20.9-36.4)	**0.016**
Waist circumference, cm	84.1 ± 1.3 (69-104)	91.4 ± 1.3 (65-109)	**0.002**
Estimated visceral fat area, cm^2^	97 ± 7 (34-192)	154 ± 8 (101-226)	**< 0.0001**
Estimated glomerular filtration rate, mL/min	74.4 ± 2.9 (34.4-114)	64.8 ± 3.3 (28.8-97.3)	**0.039**
Blood glucose, mg/dL	131 ± 46 (83-261)	130 ± 7 (84-196)	0.823
Fasting immunoreactive insulin, μIU/mL (n = 50)	8.6 ± 0.9 (2.9-15.0)	9.0 ± 1.1 (2.9-26.2)	0.860
HbA1c (NGSP), %	7.1 ± 0.1 (5.9-10.6)	7.2 ± 0.2 (5.6-9.6)	0.702
Systolic blood pressure, mmHg	130 ± 2 (105-180)	134 ± 3 (110-160)	0.708
Diastolic blood pressure, mmHg	74 ± 1 (60-100)	76 ± 1 (60-91)	0.423
Triglyceride, mg/dL	109 ± 9 (41-260)	150 ± 12 (78-396)	**0.011**
High-density lipoprotein cholesterol, mg/dL	59 ± 2 (34-97)	54 ± 2 (38-93)	0.181
Low-density lipoprotein cholesterol, mg/dL	111 ± 5 (57-208)	116 ± 6 (57-203)	0.550
Smoking (none/ex-/current-smoker)	15/13/10	9/13/6	0.604
Brinkman index	531 ± 106 (0-3000)	538 ± 124 (0-1800)	0.622
Duration of diabetes, years	10 ± 1 (1-38)	11 ± 1 (1-38)	0.529
Diabetic neuropathy	7	10	0.104
Diabetic retinopathy (NDR/SDR/PDR)	31/3/4	18/6/4	0.314
Diabetic nephropathy (stage I/II/III)	25/8/5	16/5/7	0.359
Hypertension	11	22	**0.009**
Dyslipidemia	21	21	0.100
Medications for hypertension (CA/ACEIorARB/β/diuretics/α)	7/12/3/1/1	15/18/3/2/1	0.309
Medications for diabetes (SU/BG/αGI/glinide/Insulin)	15/8/7/2/11	11/11/8/0/5	0.157
Medications for dyslipidemia (statins/fibrate)	16/0	16/0	0.125
Serum adiponectin, μg/mL (all; n = 66)	9.8 ± 0.9 (2.3-33.5)	8.4 ± 1.1 (2.2-21.5)	0.235
adiponectin, μg/mL (males)	8.5 ± 1.1 (2.3-23.0)	7.6 ± 1.2 (2.2-21.5)	0.583
adiponectin, μg/mL (females)	11.2 ± 1.5 (3.9-33.5)	9.8 ± 1.9 (4.2-11.5)	0.576
Serum interleukin-6 (IL-6), pg/mL (all; n = 66)	3.37 ± 0.39 (0.33-13.75)	2.36 ± 0.45 (0.33-5.4)	0.286
IL-6, pg/mL (males)	3.92 ± 0.59 (0.81-13.75)	2.16 ± 0.63 (0.33-5.4)	0.075
IL-6, pg/mL (females)	2.71 ± 0.46 (0.33-5.59)	2.73 ± 0.60 (0.81-4.75)	0.582
Serum TBARS, nmol/mL (all, n = 66)	4.49 ± 0.15 (2.68-7.30)	4.68 ± 0.18 (3.01-6.56)	0.423
TBARS, nmol/mL (males)	4.45 ± 0.20 (2.68-6.10)	4.44 ± 0.21 (3.01-6.56)	0.958
TBARS, nmol/mL (females)	4.53 ± 0.24 (3.24-7.30)	5.23 ± 0.35 (4.00-6.20)	0.112
FSSG scores	2.5 ± 1.4 (0-24)	8.6 ± 1.8 (0-24)	**0.015**
< 8/≥ 8	33/5	18/10	**0.031**

mean ± SEM or n (range), NDR: non-diabetic retinopathy, SDR; simple diabetic retinopathy, PDR; proliferative diabetic retinopathy, SU; sulfonyl urea, BG; biguanide, αGI; alpha glucosidase inhibitor, CA; calcium channel antagonist, ACEI; angiotensin converting enzyme inhibitor, ARB; angiotensin receptor blocker, β; β blockade, α; α blockade, TBARS; thiobarbituric acid reactive substance.

Estimated glomerular filtration rate = 194 × creatinine^-1.094 ^x age^-0.287^.

Significant level was set at p value < 0.05 (bold type).

### Contribution of the metabolic syndrome and adiponectin on GERD symptoms

**Figure 1:**
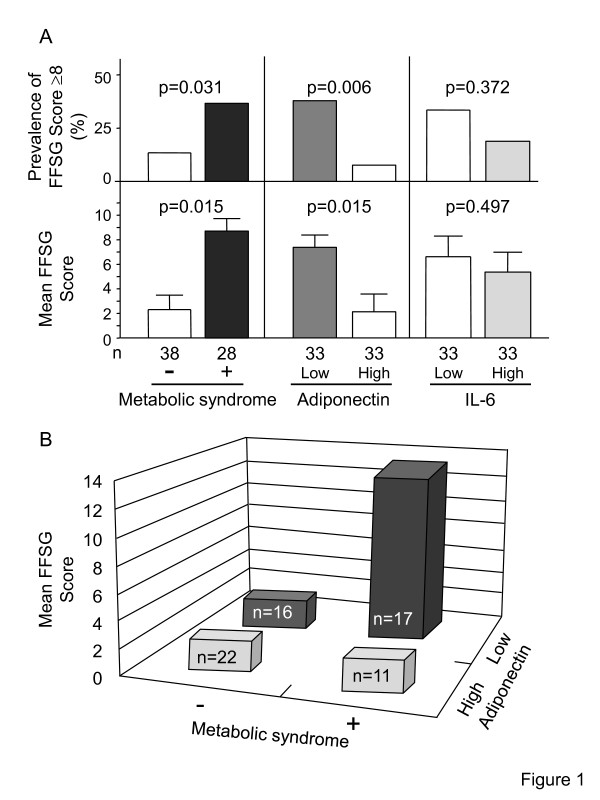
**A. Mean FSSG score in relation to the metabolic syndrome (Mets), serum adiponectin levels (serum adiponectin levels; cutoff value 6.31 μg/mL for men, 8.62 μg/mL for women, median value, respectively), and serum interleukin-6 (IL-6) (cutoff value 2.02 pg/mL for men, 2.65 pg/mL for women, median value, respectively). B. Comparison of prevalence of GERD symptoms (FSSG score ≥ 8) and FSSG score between T2DM without and with Mets, and with low and high levels of serum adiponectin**. Data are mean ± SEM, and compared by the χ^2 ^and Mann-Whitney U-test in experiments of two groups. All analyses were performed with the JMP Statistical Discovery Software 9.0 (SAS Institute, Cary, NC).

To investigate the contribution of Mets on GERD symptoms, the subjects were divided into two groups, those without and with Mets. The prevalence of FSSG score ≥ 8 and average FSSG score in T2DM subjects with Mets were significantly higher compared to those without Mets (Figure 1A). Next, to investigate the contribution of adiponectin on GERD symptoms, the subjects were divided into two groups, those with low and high circulating levels of adiponectin (cutoff value 6.31 μg/mL for men, 8.62 μg/mL for women, median value, respectively). The prevalence of FSSG score ≥ 8 and average FSSG score in T2DM subjects with low levels of serum adiponectin were significantly higher compared to those with high levels of serum adiponectin (Figure 1A). However, there was no significant difference of the prevalence of FSSG score ≥ 8 and average FSSG score in between T2DM subjects with low and high levels of serum IL-6 (cutoff value 2.02 pg/mL for men, 2.65 pg/mL for women, median value, respectively) (Figure 1A). There was also no significant difference of the prevalence of FSSG score ≥ 8 and average FSSG score in between T2DM subjects with low and high levels of serum TBARS (cutoff value 4.24 nmol/mL for men, 4.90 nmol/mL for women, median value) (27% versus 21%; p = 0.565, 6.0 ± 1.7 versus 4.9 ± 1.7; p = 0.648, data no shown).

Finally, multiple regression analysis (adopted factors; age, sex, Mets, adiponectin, Mets × adiponectin) identified interaction term (Mets × adiponectin) as a significant determinant of GERD symptom score (p = 0.047, Figure 1B). These results indicated that the combination of Mets and hypoadiponectinemia had a multiplicative effect on GERD symptom score.

## Discussion

The study of Japanese T2DM patients demonstrated for the first time that 1) the prevalence of T2DM with FSSG score ≥ 8 was 23% (n = 15/66), 2) the coexistence of Mets and low levels of serum adiponectin was associated with GERD symptoms. We have demonstrated that adiponectin suppresses inflammation in various organs, such as the heart, lung, aorta, kidney, liver, colon and pancreas [23]. Adiponectin may also play a protective role of erosive esophagitis. To clarify the protective effect of adiponectin on GERD, further experimental studies are required. On the other hand, increased systemic IL-6 concentrations are associated with the pathophysiology of T2DM, with adipose tissue being the major source of this cytokine [24]. Exposure to components of the gastric refluxate is sufficient to stimulate esophageal cells to release a pro-inflammatory cytokine, IL-6, with the potential to mediate the esophageal motor abnormalities associated with GERD-induced esophagitis [25]. However, the present study show serum IL-6 levels are not associated with GERD symptoms in T2DM. Taken together, the results suggest that decreased anti-inflammatory cytokines rather than increased pro-inflammatory cytokines may be associated with the development of GERD-induced erosive esophagitis. We described here the prevalence and characteristics of Japanese diabetics with GERD symptoms, and it may be therefore necessary to diagnose a treatable GERD from the standpoint of prevention of lifestyle-related diseases as well as improvement of QOL in T2DM patients. Large-scale interventional trials, such as weight reduction, intensive anti-GERD and anti-diabetes (especially thiazolidinedione which is known to increase serum adiponectin) drugs or combinations of these therapies, should be provided to assess the effects of appropriate treatment on the outcome of T2DM patients with GERD symptoms.

Several limitations of this study must be considered. First, this is a cross-sectional study, making it difficult to establish a cause-effect relationship. Further prospective studies should be conducted in the future to analyze this relationship. Second, the results may not be applicable to females or non-Japanese populations. Third, the current study did not include the effects of alcohol intake, smoking habits, mental status, dietary habit and use of pharmacotherapy (such as nonsteroidal anti-inflammatory drugs; NSAIDs). The current study found that the prevalence of FSSG score ≥ 8 and average FSSG score were not influenced by smoking habits and use of NSAIDs. Finally, further replication studies of larger sample need to be designed including these confounding variables, such as potential factors to be influenced serum adiponectin levels, such as smoking status, use of pharmacotherapy (ACE-I/ARB, statin), in the future.

In conclusion, the coexistence of MetS and low levels of serum adiponectin was associated with the higher prevalence of GERD symptom in subjects with T2DM.

## List of Abbreviations

BIA: bioelectrical impedance analysis; CV: coefficient of variation; eVFA: estimated visceral fat area; FSSG: Frequency Scale for the Symptoms of GERD; GERD: gastroesophageal reflux disease; IL-6: interleukin-6; Mets: metabolic syndrome; QOL: quality of life; TBARS: thiobarbituric acid-reacting substance; T2DM: type 2 diabetes mellitus.

## Competing interests

K.K. and T.F. are members of the "Department of Metabolism and Atherosclerosis", a sponsored course endowed by Kowa Co. Ltd. and a company researcher is dispatched to the course. All other authors declare no competing interests.

## Authors' contributions

A.H. and K.K. researched and analyzed data. K.K. also participated in the concept and design of the study, interpretation of data and reviewed/edited the manuscript. H.N., K.I. and A.H-S. recruited the patients and collected the data. T.F. and I.S. contributed to discussion and wrote the manuscript. All authors read and approved the final version of the manuscript.
